# Inter-layer and inter-subject variability of diurnal gene expression in human skin

**DOI:** 10.1093/nargab/lqac097

**Published:** 2022-12-31

**Authors:** Marta del Olmo, Florian Spörl, Sandra Korge, Karsten Jürchott, Matthias Felten, Astrid Grudziecki, Jan de Zeeuw, Claudia Nowozin, Hendrik Reuter, Thomas Blatt, Hanspeter Herzel, Dieter Kunz, Achim Kramer, Bharath Ananthasubramaniam

**Affiliations:** Institute for Theoretical Biology – Laboratory of Theoretical Chronobiology, Humboldt Universität zu Berlin and Charité Universitätsmedizin Berlin, Philippstraße 13, House 4, 10115 Berlin, Germany; Research and Development, Beiersdorf AG, 20245 Hamburg, Germany; Institute for Medical Immunology – Laboratory of Chronobiology, Charité Universitätsmedizin Berlin, Charitéplatz 1, 10117 Berlin, Germany; Institute for Medical Immunology – Laboratory of Chronobiology, Charité Universitätsmedizin Berlin, Charitéplatz 1, 10117 Berlin, Germany; Berlin Institute of Health – Center for Regenerative Therapies (BCRT), Charité Universitätsmedizin Berlin, Augustenburger Platz 1, 13353 Berlin, Germany; Department of Infectious Diseases and Respiratory Medicine, Charité Universitätsmedizin Berlin, Charitéplatz 1, 10117 Berlin, Germany; Institute for Medical Immunology – Laboratory of Chronobiology, Charité Universitätsmedizin Berlin, Charitéplatz 1, 10117 Berlin, Germany; Institute of Physiology – Sleep Research & Clinical Chronobiology, Charité Universitätsmedizin Berlin, Charitéplatz 1, 10117 Berlin, Germany; Institute of Physiology – Sleep Research & Clinical Chronobiology, Charité Universitätsmedizin Berlin, Charitéplatz 1, 10117 Berlin, Germany; Research and Development, Beiersdorf AG, 20245 Hamburg, Germany; Research and Development, Beiersdorf AG, 20245 Hamburg, Germany; Institute for Theoretical Biology – Laboratory of Theoretical Chronobiology, Humboldt Universität zu Berlin and Charité Universitätsmedizin Berlin, Philippstraße 13, House 4, 10115 Berlin, Germany; Institute of Physiology – Sleep Research & Clinical Chronobiology, Charité Universitätsmedizin Berlin, Charitéplatz 1, 10117 Berlin, Germany; Institute for Medical Immunology – Laboratory of Chronobiology, Charité Universitätsmedizin Berlin, Charitéplatz 1, 10117 Berlin, Germany; Institute for Theoretical Biology – Laboratory of Theoretical Chronobiology, Humboldt Universität zu Berlin and Charité Universitätsmedizin Berlin, Philippstraße 13, House 4, 10115 Berlin, Germany; Institute for Theoretical Biology – Laboratory of Systems Chronobiology and Chronomedicine, Humboldt Universität zu Berlin, Philippstraße 13, House 20, 10115 Berlin, Germany

## Abstract

The skin is the largest human organ with a circadian clock that regulates its function. Although circadian rhythms in specific functions are known, rhythms in the proximal clock output, gene expression, in human skin have not been thoroughly explored. This work reports 24 h gene expression rhythms in two skin layers, epidermis and dermis, in a cohort of young, healthy adults, who maintained natural, regular sleep-wake schedules. 10% of the expressed genes showed such diurnal rhythms at the population level, of which only a third differed between the two layers. Amplitude and phases of diurnal gene expression varied more across subjects than layers, with amplitude being more variable than phases. Expression amplitudes in the epidermis were larger and more subject-variable, while they were smaller and more consistent in the dermis. Core clock gene expression was similar across layers at the population-level, but were heterogeneous in their variability across subjects. We also identified small sets of biomarkers for internal clock phase in each layer, which consisted of layer-specific non-core clock genes. This work provides a valuable resource to advance our understanding of human skin and presents a novel methodology to quantify sources of variability in human circadian rhythms.

## INTRODUCTION

The skin is the largest organ of the body and its main functions are to protect against bacteria, radiation or temperature from the exterior, as well as against water loss from the interior (1). It is morphologically complex and consists of many cell types that are organized into three main layers: epidermis, dermis and hypodermis (2). The skin evolved a circadian clock (3) in response to the direct exposure to the rhythmic external environment to anticipate changes and to adjust its physiology accordingly.

The mammalian circadian clock is a hierarchical network with the central clock in the suprachiasmatic nucleus (SCN) and peripheral clocks in many tissues including the skin. The cell-autonomous molecular ‘core’ clock (4,5) consists of a number of interlocked transcriptional-translational negative feedback loops. Core clock genes *CLOCK* and *ARNTL* induce the expression of their own inhibitors, *PER* and *CRY* genes. Once translated, PER and CRY proteins form large complexes that travel back to the nucleus to repress CLOCK and ARNTL, thus repressing their own transcription and thereby generating self-sustained 24 h rhythms in gene and protein expression. In mammals, core clock components that are also transcription factors act at *cis*-regulatory sequences to drive rhythmic expression of a large number of output genes (about 10% of all genes) in a cell-autonomous and tissue-specific manner (6,7).

The circadian gene expression profile of the skin remains nevertheless incompletely characterized. The presence of a skin circadian clock in humans was first inferred from circadian rhythms in biophysical skin parameters, such as sebum secretion (8), water loss (9) and response to allergens (10). At the turn of the century, rhythmic expression of selected core clock genes in the skin was described in humans (11) and mice (12). Over time, circadian expression of core clock genes was recorded in several skin cell types, including epidermal and hair follicle keratinocytes, dermal fibroblasts and melanocytes (13–17). Spörl *et al.* (18) performed the first high-throughput analysis of 24 h gene expression rhythms in the skin under natural light-dark conditions; henceforth termed ‘diurnal’ gene expression. That study identified ∼300 diurnal genes from measurements at three time points in one layer (epidermis). More recently, Wu et al. (19,20) identified ∼100–150 diurnal genes each in the epidermis and dermis from samples collected every 6  h over one day. However, both these microarray studies provide only limited insight into rhythmic 24 h gene expression in human skin, since they lacked sufficient number of samples over one 24 h cycle. To more thoroughly describe the influence of the human skin clock, we identified diurnal genes in the two prominent skin layers, epidermis and dermis. To assess whether the complex and heterogeneous skin also results in a cell type-/layer- specific clock, we compared diurnal gene expression across layers.

As one of the few accessible tissue clocks, skin samples could be used for circadian phenotyping of humans. Evidence is gradually accumulating that therapeutic efficacy and the degree of side effects are dependent on the time of drug administration (21–23). Such observations are likely to grow, since 50% of all drugs target the product of a circadian gene (6,7). One key challenge to implementing time-of-day-aware ‘circadian medicine’ strategies is the fact that internal clocks of humans are heterogeneous. Since rhythms in human physiology are determined by *internal* clock time and not on time according to the external environment, circadian studies in humans ought to record and present results relative to the internal phase of entrainment (termed chronotype) of subjects. This internal clock time in turn depends on genetic factors (16,24), age (25), sex (25), level of light exposure (26,27), the season (26,28) and on the local time-zone (25). Thus, circadian treatments need to be personalized to a subjects’s clock. To evaluate the utility of skin samples for circadian phenotyping, we used our comprehensive gene expression profiles to identify biomarkers for circadian phase in each layer.

We measured gene expression using microarrays in a small cohort of young, healthy adults of both sexes, who maintained their natural yet regular sleep schedules. We first quantified the diurnal gene expression expected in the general population in the epidermis and dermis. In contrast to previous studies on skin, we present our results with respect to internal time of subjects; our study design included chronotyping of subjects. We then analyzed the layer-specificity of *population* diurnal rhythms. The population diurnal rhythms are the most representative rhythms of an individual in the population. However, the inherent heterogeneity will result in individual rhythms diverging from the population rhythm. Therefore, we next quantified how much individual diurnal rhythms deviate from the population rhythms in both layers. Finally, we identified a small set of biomarkers with diurnal gene expression to predict internal clock phase in each layer.

## MATERIALS AND METHODS

### Study design

This study was conducted in March and April 2011 at the Clinic for Sleep and Chronomedicine at the St. Hedwig Hospital Berlin. The 15-day study consisted of saliva sample collection for dim light melatonin onset (DLMO) assessment on nights 6, 7, 13 and 14 followed by skin sample collection on day 15 every 4 h for 24 h for gene expression quantification under regular entrained living conditions. Saliva samples to determine cortisol and melatonin levels were also collected concurrently with the skin sample (hormone profiles are shown in Supplementary Figure S1).

### Study protocol

Participants had to keep their self-selected regular sleep-wake cycles (and accompanying habits) during the entire 15-day period with sleep hygiene and regularity monitored using actimetry (Actiwatch type 4, Cambridge Neurotechnology, UK). On the evenings of days 6, 7, 13 and 14, participants visited the laboratory to provide saliva samples every half hour from 19:30 to 23:00 for DLMO assessment.

In preparation for skin sampling, participants slept the night of day 14 in the sleep laboratory, where they were provided their own room and bathroom. On day 15, they were woken up at 7:30 and beginning at 8:00, one skin punch (3 mm diameter) was taken from the lower back every 4 h over a 24 h period (8:00, 12:00, 16:00, 20:00, 0:00, 4:00, 8:00). Participants remained in regular room lighting for the rest of the day, which was not strictly controlled. They were allowed to move around as they pleased and were not in time isolation. Equicaloric meals were provided. After the 0:00 sample, participants went to sleep in complete darkness (0 lx). At 4:00, they were briefly woken up for a skin biopsy after which they slept until 7:30. At 8:00, the last biopsy sample was taken and participants were free to leave. The biopsy was separated into epidermis and dermis and frozen for subsequent gene expression analyses. Wound care was performed by suturing and wound dressing. All samples were processed at Beiersdorf AG and the remaining material was destroyed.

### Study approval

The study to obtain human skin biopsies was approved by the local Ethical Review Board at Charité Universitätsmedizin Berlin (EA4/019/11). Tissue samples were collected according to the recommendations of the Declaration of Helsinki and to applicable laws for a non-drug study. All donors provided written and informed consent.

### Participants

Eleven healthy volunteers (six males, five females, aged 20–30 years in 2011) with intermediate chronotypes and self-selected habitual bedtimes between 22:00 and 24:00 were recruited for the study. Chronotypes were assessed using Munich Chronotype Questionnaire (MCTQ) by calculating the mid sleep time on free days adjusted for the sleep-debt accumulated during the workweek MSF_sc_ (29). Participants were free of any medical, psychiatric or sleep disorder, did not engage in shift work and did not experience jet-lag in the 3 months preceding the study. Moreover, volunteers who displayed unusual light reaction of the skin, were intolerant to anesthetics, had tattoos or scars in the test area, had unusual scarring of the skin, had received radiation therapy in the past two years, used self-tanning products in previous 2 weeks and/or showed signs of drug, alcohol or nicotine abuse were excluded from the study.

All information about the study participants is provided in Supplementary Table S1.

### Skin sample processing

Skin biopsies were subsequently incubated in PBS at 55°C for 3 min to separate the epidermis and the dermis. Tissue samples were then frozen in liquid nitrogen and stored at −80°C. RNA extraction and quality control from skin biopsies was performed by Miltenyi Biotec using the TRIzol method. Linear amplification and labeling of RNA and hybridization of Agilent Whole Genome Oligo Microarrays 4 × 44k (Agilent Technologies) using 1.2–1.65 µg of Cy3-labeled cRNA was performed by Miltenyi Biotec, essentially as reported in (30).

### Gene expression analysis

The microarray gene expression analysis was conducted in R. The RMA (Robust Multichip Average) algorithm was used to pre-process and extract expression profiles from the raw CEL files. Genes were annotated with ENSEMBL and Entrez IDs using Agilent ‘Human Genome, Whole’ annotation data (hgug4112a.db, v3.2). The raw gene expression data has been deposited in the Gene Expression Omnibus (GEO) under the accession number GSE205155.

Raw data of the hybridized microarrays were normalized and processed using the Bioconductor R-Project package Linear Models for Microarray Data (limma). Probes with spot intensities above background in half of the 154 samples were retained for further analysis as ‘expressed’. This number was chosen for two reasons: (i) to approximately retain probes expressed in at least one layer (since we have paired samples across layers) and (ii) to have sufficiently many samples for rhythm assessment in each layer across heterogeneous human subjects. Background correction was performed as suggested by the limma user guide. From the 62976 total probes, this filtering resulted in 39703 ‘expressed’ probes. In successive filtering steps, control probes (3951) and probes without gene annotations (hgug4112a.db) were removed, leaving 24135 ‘expressed’ probes mapped to genes. Finally, probes mapped to the same gene were averaged, resulting in a final list of 11578 expressed genes. This is the standard pipeline recommended for microarray analysis in the limma user guide.

Principal component analysis (PCA) was performed to analyze the sources of variation in the dataset. It showed both the difference between tissues as the greatest source of variation and the outlier sample (E32_P109) that was removed from the analysis (Supplementary Figure S2A).

### Rhythmicity analysis

To detect genes exhibiting diurnally-rhythmic behavior with a 24 h period in their expression, we used cosinor analysis and differential rhythmicity analysis, which is based on a cosinor/sine-fitting approach, as described in (31). In short, we first tested the null hypothesis that the sine and cosine terms from cosinor analysis are equal to 0. Acrophases and amplitudes were estimated from the analysis and used to identify significantly oscillating genes. If the null hypothesis could be rejected under a false discovery rate (FDR) threshold < 0.05 and a minimum amplitude requirement (i.e., that either the amplitude of the oscillating gene in dermis *or* in epidermis is above a peak-to-trough fold change amplitude > 1.5) was satisfied, we classified that gene as diurnally-rhythmic in at least one of the layers. Next, we tested the differential rhythmicity null hypothesis, namely that, among the genes that were rhythmic in at least one layer, sine and cosine terms are equal across skin layers. If this hypothesis could be rejected under a FDR < 0.05, the gene was considered to have significantly *different* diurnal rhythms in dermis compared to epidermis. If, on the contrary, the null hypothesis could not be rejected, we defined the gene to have *indistinguishable* rhythms across layers. Importantly, genes with amplitudes below the threshold in one of the layers but above the threshold in the other layer (that passed the minimum amplitude requirement), and with statistically indistinguishable rhythms, were considered rhythmic and were included in our analyses despite the amplitude value being below the threshold in one layer in the first step of the analysis.

In analyses where *internal* time was used, sampling (wall) time was corrected to internal time in each subject by subtracting the mid-sleep time on free days after correcting for sleep debt during week days (MSF_sc_) (29) to wall time. The adjustment of wall time to the individual’s chronotype resulted in the intervals between timepoints being no longer fixed, meaning that state-of-the-art non-parametric methods for rhythm detection that need uniform samples (JTKcycle or RAIN) could not be used.

### Functional annotation and gene phase set enrichment analysis (PSEA)

Genes showing significant rhythmic expression patterns in one skin layer as well as those genes with significant/non-significant differential diurnal rhythms across layers were tested for over-representation in Reactome pathways against the background of expressed genes or diurnal genes in the skin using the ReactomePA package (32). Pathways containing fewer than 20 diurnal genes were excluded from the analyses. The backgrounds used for each analysis have been specified in the respective figure captions.

Phase Set Enrichment Analysis (33) was performed to further investigate the synchrony of pathways. Gene sets were downloaded from the Molecular Signatures database (MSigDB) C2 (REACTOME gene sets) (34). Sets containing fewer than five diurnal genes were excluded from the analysis. The Kuiper test was used to identify diurnally-rhythmic gene sets by comparing the acrophases of all rhythmic genes (rounded to the full hour) belonging to each gene set to a uniform background distribution and by testing for nonuniformity (*q* < 0.05).

### Assessment of variability in rhythmic parameters across subjects and skin layers

In order to analyze how magnitudes, amplitudes and phases of individual diurnal genes vary across subjects and layers, we analyzed each gene separately using linear mixed models (35–37). The expression of gene *i*, *g*_*i*_, is modeled as

(1) $$\begin{eqnarray*} g_i(t) &=& (m_i + \Delta m_{i,subj} + \Delta m_{i,layer}) \nonumber \\ && + \, (a_i + \Delta a_{i,subj} + \Delta a_{i,layer}) \cos \omega t \nonumber \\ && + \, (b_i + \Delta b_{i,subj} + \Delta b_{i,layer}) \sin \omega t + \epsilon , \end{eqnarray*}$$

where *m*_*i*_, *a*_*i*_ and *b*_*i*_ represent the coefficients of the fixed effects for gene *i*; and Δ*m*_*i*_, Δ*a*_*i*_ and Δ*b*_*i*_ represent the random effects attributed to differences across layers or subjects, which are drawn from a normal distribution, whose variance is estimated, and ε is random noise. In other words, the average expression of gene *i* is

$$\begin{equation*} \mathrm{E}(g_i(t)) = m_i + a_i \cos \omega t + b_i \sin \omega t. \end{equation*}$$

The expression of gene *i* in a particular layer in a certain subject is a random deviation from this average expression with additive contributions from layer *u*_layer_ and subject *u*_subj_:

(2) $$\begin{eqnarray*} g_i(t) &=& \mathrm{E}(g_i) + [1,\cos \omega t, \sin \omega t] \, u_\mathrm{layer} \nonumber \\ && +\, [1,\cos \omega t, \sin \omega t] \, u_\mathrm{subj} + \epsilon . \end{eqnarray*}$$

The random deviations are drawn from a normal distribution with separate covariances for subject and layer:

$$\begin{eqnarray*} && \Sigma _{i,\rm {subj}} = \rm {Cov}[u_\mathrm{subj}]=\mathrm{Cov}\begin{bmatrix}\Delta m_{i,\mathrm{subj}} \\ \Delta a_{i,\mathrm{subj}} \\ \Delta b_{i,\mathrm{subj}} \end{bmatrix} \, \, \nonumber \\ && \Sigma _{i,\rm {layer}} = \rm {Cov}[u_\mathrm{layer}]= \mathrm{Cov}\begin{bmatrix}\Delta m_{i,\mathrm{layer}} \\ \Delta a_{i,\mathrm{layer}} \\ \Delta b_{i,\mathrm{layer}} \end{bmatrix}. \end{eqnarray*}$$

To ensure tractability, we restrict the latter covariance to be diagonal, thus requiring fewer parameters to be estimated.

The gene’s average magnitude *m*_*i*_ and the variability therein attributed to layers/subjects are directly available from the mixed model fits (Equation 2). However, amplitude *A*_*i*_ and phase ϕ_*i*_ of a gene *i* are functions of the coefficients *a*_*i*_ and *b*_*i*_ given by $A_i=\sqrt{a_i^2 + b_i^2}$ and $\phi _i=\arctan \frac{b_i}{a_i}$. Variability in amplitude and phase across subjects and layers can be computed using error propagation, a classic approach from experimental physics (38). The variance of amplitude and phase across subjects and layers (*$x$* = layer, subj) can be computed from the Jacobian matrices $\mathbb {J}_{i,x}$ and the covariance matrices $\Sigma_{i,x}$ of the rhythm parameters as

$$\begin{equation*} \sigma ^2_{A,i,x} = \mathbb {J}_{A,i,x} \, \Sigma _{i,x} \, \mathbb {J}_{A,i,x}^\mathrm{T} \, \rm {and} \, \sigma ^2_{\phi ,i,x} = \mathbb {J}_{\phi ,i,x} \, \Sigma _{i,x} \, \mathbb {J}_{\phi ,i,x}^\mathrm{T}, \end{equation*}$$

where $\mathbb {J}_{A,i,x}=\left( \frac{\partial A_i}{\partial m_{i,x}} \ \frac{\partial A_i}{\partial a_{i,x}} \ \frac{\partial A_i}{\partial b_{i,x}} \right)$ (and $\mathbb {J}_{\phi ,i,x}=\left( \frac{\partial \phi _i}{\partial m_{i,x}} \ \frac{\partial \phi _i}{\partial a_{i,x}} \ \frac{\partial \phi _i}{\partial b_{i,x}} \right)$). Error propagation has been previously employed in a simpler cosinor problem to estimate noise in the estimates of amplitude and phase (39). That approach (using linear regression) does not attempt to model the different correlation structures that arise in longitudinal data across two layers.

### Identification of predictive biomarkers of molecular skin phase

We used ZeitZeiger (40) to identify skin biomarkers of circadian phase. We tested two sets of predictors using the whole set of expressed genes in epidermis or dermis separately. The predicted variable was, in both cases, internal time. To evaluate the performance of the predictors, we followed a leave-one-subject-out cross-validation approach in the lines of (40,41). To do this, predictors are trained with data from all subjects except one and internal time from the subject who is left-out is predicted. The process is iterated along all subjects and for different values of the two main parameters of ZeitZeiger, *sumabsv* and *nSPC*. The first parameter *sumabsv* controls how many genes form each sparse principal component (SPC) and the second parameter, *nSPC*, controls how many SPCs are used for prediction. Large values of either parameter result in more genes being needed for prediction. For each set of values of *sumabsv* and *nSPC* from the leave-one-subject-out cross-validation, we calculated the median absolute difference between the predicted and the observed internal time stamp across all subjects. We refer to this metric as median absolute error (MAE), and it serves as a measure of accuracy of the prediction: the lower the error, the better the prediction.

## RESULTS

### Only a third of population diurnal genes are layer-specific in healthy human skin

**Figure 1.**
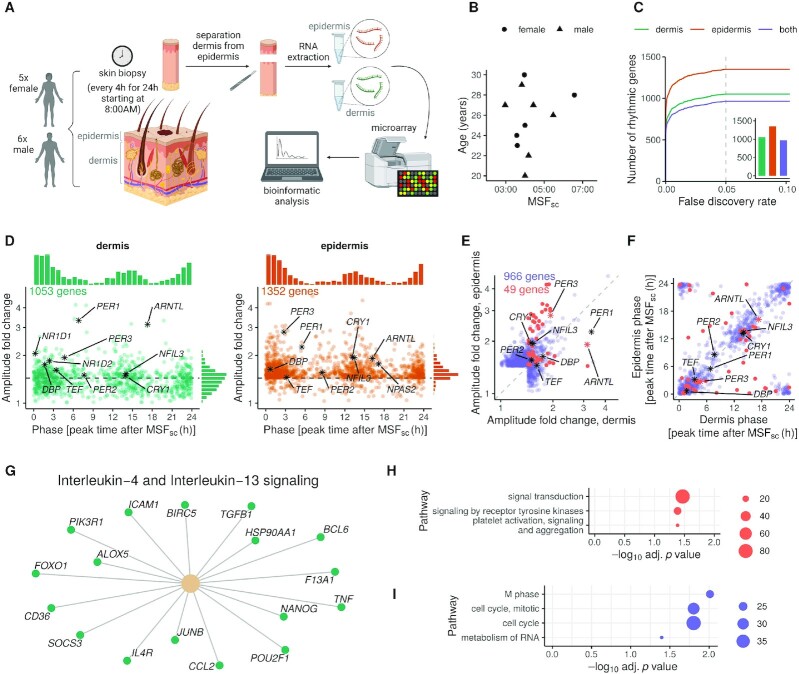
Functional and layer-specific clocks in human dermis and epidermis. (**A**) Experimental setup: 11 healthy subjects were biopsied in the back every 4 h for 24 h starting at 8:00. Dermis and epidermis were separated and gene expression was analyzed using whole-genome microarrays. (**B**) Composition of the study cohort by sex, age and mid-sleep time on free days after correcting for sleep debt (MSF_sc_). (**C**) Number of diurnally-rhythmic genes with respect to *internal* time in dermis, epidermis or in both layers were determined using differential rhythmicity analysis (minimum requirement of peak-to-trough fold change amplitude >1.5 in at least one layer, see Materials and Methods for details). The number of genes for FDR < 0.05 is shown in the inset. (**D**) Phase (as peak time after MSF_sc_) and amplitude distributions of the diurnal genes in human dermis (in green, left panel) and epidermis (orange, right panel) (FDR < 0.05, peak-to-trough fold change amplitude > 1.5). Each gene is represented by a dot; clock genes are highlighted in black. (**E**) Amplitude correlation of the 966 diurnal genes in both layers, from which 49 show significantly different rhythms (highlighted in coral). (**F**) Phase correlation of diurnal genes in dermis *and* epidermis, with differentially rhythmic genes highlighted in coral. Clock genes are shown in black asterisks (or coral, if differentially rhythmic). (**G**) Reactome pathway enrichment analysis of the 1053 diurnal genes in dermis tested against the background of all 11578 expressed genes. (**H**) Reactome pathway enrichment analysis of the 522 differentially-rhythmic genes in dermis and epidermis tested against the background of all 1439 genes rhythmic in dermis or epidermis. (**I**) Reactome pathway enrichment analysis of the 917 genes with indistinguishable rhythms in dermis and epidermis tested against the background of all 1439 genes diurnally-rhythmic in dermis or epidermis. Only pathways containing more than 20 genes per set at a *P* < 0.05 are shown.

To explore molecular diurnal rhythms in human dermis and epidermis, 11 healthy subjects (male and female) were biopsied in the lower back every 4  h across a 24  h duration (Figure 1A, Materials and Methods). In the two weeks leading up to the biopsies, subjects maintained their desired natural sleep-wake schedule. Biopsies were separated into dermis and epidermis and subsequently quantified using whole-genome microarrays. We adjusted sample collection times using their chronotypes to indicate *internal* time of subjects (Figure 1B). Chronotypes were estimated using the Munich Chronotype Questionnaire (MCTQ) (Supplementary Table S1) as the mid-sleep time on free days after correcting for sleep debt (MSF_sc_) (29). We attempted in our study to assess chronotypes by means of the saliva dim light melatonin onset (DLMO) marker (42), which is considered the gold-standard for internal clock time (43), but technical difficulties limited our DLMO assessment to six subjects.

The samples highly expressed marker genes for the corresponding skin layer. Principal component analysis revealed that inter-layer differences was the greatest source of variation among the samples (Supplementary Figure S2A). Differential expression (DE) analysis identified 1976 genes in dermis with at least two-fold over-expression relative to epidermis and 1164 genes in epidermis with at least 2-fold over-expression relative to dermis (Supplementary Figure S2B, Table S2). The DE genes in dermis are involved in extra-cellular matrix (ECM) organization and collagen formation, according to Reactome pathway enrichment, consistent with the dermis’ role as underlying connective tissue in the skin (44) (Supplementary Figure S2C). The DE genes in the epidermis are highly enriched for keratinzation and formation of cornified envelope, both of which are hallmarks of the epidermis (45) (Supplementary Figure S2C). Among the top 10 DE genes in the dermis compared to epidermis, we found *DCD*, an antimicrobial peptide, important for the innate immune system known to be expressed in eccrine glands in the dermis (Human Protein Atlas (46)), while *FLG, CDSN* and *KLK5*, which are expressed in the strateum corneum and granulosum in the epidermis (Human Protein Atlas), were found in the top 50 DE genes in the epidermis.

Two-thirds of the diurnal genes have similar rhythms of gene expression in both layers. We identified and compared genes with diurnal population rhythms in both human skin layers using differential rhythmicity analysis (31). *Population* rhythms are diurnal patterns of gene expression averaged across the entire cohort. We identified 1053 diurnal genes in dermis and 1352 in epidermis (FDR < 0.05 and amplitude >1.5-fold peak-to-trough in at least one layer, see Materials and Methods for details; Supplementary Table S3). 966 of these diurnal genes were common to both skin layers (Figure 1C, inset, Supplementary Figure S3A). The number of rhythmic genes remained stable across a range of choices of FDR cutoff (Figure 1C). 386 genes were rhythmic only in the epidermis and 87 only in the dermis, as well as a further 49 genes that were rhythmic in both but with significantly different amplitude and/or phase. Thus, the expression of 917 diurnal genes was indistinguishable between the two layers.

Diurnal gene amplitudes are larger in the epidermis, but core clock genes are remarkably similar in the two layers. We observed a bimodal distribution of phases of all diurnal dermal and epidermal genes, with peaks clustering at 1 h and 13 h after MSF_sc_ (Figure 1D and Supplementary Figure S3A). Despite similar phase distributions, amplitudes of individual genes differed between layers. The common (966) diurnal genes in the epidermis cycled with a larger amplitude than in the dermis (Wilcoxon signed rank test, *P* < 0.0001). Among these, differentially-rhythmic genes possessed significantly larger epidermal amplitudes (95% CI of difference = (0.11, ∞)), while the 917 genes with similar rhythms across layers showed a trend in this direction (Figure 1E). There was no systematic difference in the phase of diurnal genes common to the two layers (Figure 1F, mean phase difference = –0.08 h, *P* < 0.0001, Rayleigh test of resultant vector *R* = 0.85). The core clock genes were remarkably consistent (statistically indistinguishable) in amplitude and phase between the two layers (Supplementary Figure S3B), i.e., they were not called differentially-rhythmic in our analysis. The only exceptions were *ARNTL* and *PER3*, which had larger amplitudes in the dermis and epidermis, respectively. Note, *NR1D1* and *NR1D2* were rhythmic in both layers but with amplitudes just outside the amplitude cutoff in the epidermis.

The similar and dissimilar rhythms in the two layers are involved in cell cycle and cellular signaling, respectively. Diurnal genes in the dermis were enriched for immune response-related pathways; in particular, multiple analyses (Reactome, KEGG, MSigDB) revealed enrichment of IL-4 and IL-13 signaling pathways (Figure 1G). However, diurnal genes in the epidermis did not show any statistically significant enrichment. The differentially-rhythmic genes (diurnal genes with dissimilar rhythms in the two layers) were primarily involved in three pathways: platelet activation & signaling, signal transduction and signaling by receptor tyrosine kinases (Figure 1H). On the other hand, 917 genes with indistinguishable rhythms in the two layers were highly enriched among all rhythmic genes for cell cycle genes especially relating to the mitosis phase (Figure 1I). To investigate further the synchrony of pathways within these bimodal peaks (Figure 1D), we performed Phase Set Enrichment Analysis (33) of the rhythmic genes in the two layers against a background of uniform phase distribution. Surprisingly, the highly phase synchronized rhythmic pathways in both dermis and epidermis concerned DNA repair, RNA processing, and Unfolded Protein Response (Supplementary Figure S3C).

Diurnal genes in this study had limited overlap with prior studies. We compared our list of rhythmic genes with previous human studies that quantified gene expression rhythms from time-series data in the epidermis (19), dermis (20) and hair-follicle cells (47), which lie both in the dermis and epidermis (Supplementary Table S3). Little less than half the rhythmic genes (epidermis: 54/188, dermis: 24/59) identified by Wu *et al.* (19,20) with a 6 h sampling resolution overlapped with our gene lists. Similarly, our study (dermis and epidermis combined) shared merely 22 of the 251 rhythmic genes in hair-follicle cells. The amplitudes and phases of overlapping diurnal genes too correlated poorly with our estimates (Supplementary Table S3).

Population diurnal rhythms are not affected by choice of time reference in this dataset. We repeated the above analysis without correcting the sample collection times for subject chronotypes using MSF_sc_. Interestingly, the rhythmic genes determined with respect to external time (Supplementary Figure S4) did not deviate appreciably in number, amplitude or phase from the diurnal genes (compare Figure 1C–F and Supplementary Figure S4) with respect to internal time, i.e, controlling for MSF_sc_ did not affect rhythmicity analysis significantly in our dataset.

In summary, we observed significant similarity in the diurnal gene expression across these two adjacent skin layers complemented by some layer-specificity of diurnal rhythms.

### Amplitudes and phases of diurnal genes are subject-specific, while magnitudes are layer-specific

**Figure 2.**
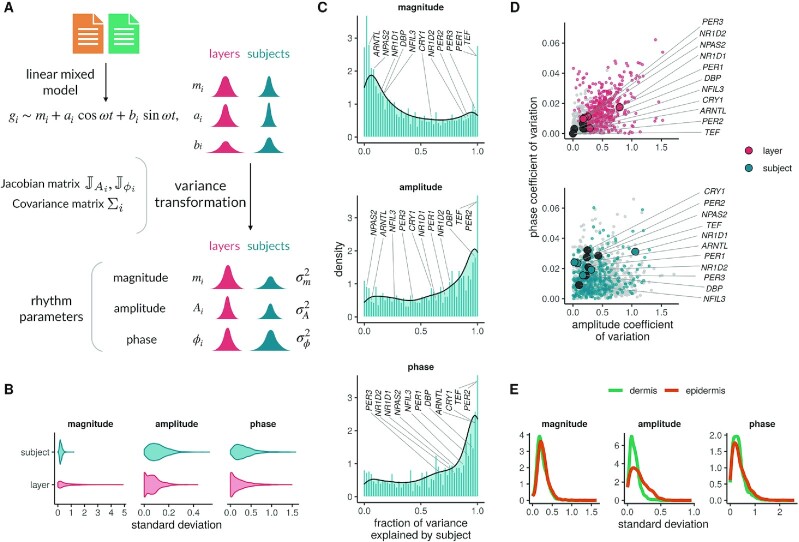
Variability of diurnal gene expression across layers and subjects. (**A**) Methodology to compute variability in rhythmic parameters across layers and subjects. (**B**) Quantification of the variability in magnitude (MESOR), amplitude and phase across subjects (blue) and layers (red) in genes (1439) diurnally-rhythmic in at least one layer. (**C**) Relative contribution of layer and subject to variation in magnitudes, amplitudes and phases. The fraction of variance explained by subject was defined as $\sigma ^2_\rm {subj}/(\sigma ^2_\rm {subj} + \sigma ^2_\rm {layer})$. Diurnal genes falling in bins closer to 0 represent rhythmic genes with low fraction of variance attributed to subject differences (and high fraction of variance explained by layer differences). (**D**) Correlations of amplitude and phase relative variability (using the coefficient of variation) across layers (top panel) or subjects (bottom panel). Clock genes are shown with larger dots. Differentially-rhythmic genes (from the previous analysis) across layers are shown in color (red, blue), while genes with non-significant rhythm differences in dermis and epidermis are shown in grey. (**E**) Quantification of the variability in rhythmic parameters across subjects in each skin layer separately. Linear mixed models were fit to all 1439 diurnal genes rhythmic in at least one layer for plot panels B–D; in all 1053 rhythmic genes in dermis and 1352 in epidermis for plot panel E.

The population (mean) diurnal gene expression describes rhythmic gene expression at the level of the cohort. We quantified next how much rhythmic parameters of the diurnal genes varied among individuals in the cohort. We present this variability within the cohort in relation to the variability across layers. We fit linear mixed models (37) followed by error propagation to obtain both the average diurnal gene expression (fixed effects) and the variation across subjects and layers (random effects) (Figure 2A). This analysis was performed only on the 1439 genes that showed population rhythms in at least one layer. We computed the variability in diurnal parameters (magnitude (MESOR), amplitude and phase) of individual rhythmic genes across both layers and subjects (Supplementary Table S4).

Magnitudes vary more across layers, and amplitudes and phases more across subjects. The error propagation analysis produced estimates of the absolute variability (as standard deviations) of the rhythmic parameters across subjects and layers. When the variability is viewed in absolute terms (Figure 2B), magnitude of diurnal genes varied more across layers than across subjects, while amplitudes and phases were more variable across subjects than across layers. In order to better quantify the relative contribution of layer and subject to the diurnal rhythm variability, we defined the fraction of variance explained by subject as $\sigma ^2_\rm {subj}/(\sigma ^2_\rm {subj} + \sigma ^2_\rm {layer})$. More genes were variable in amplitude and phase across subjects (have fraction of variance explained closer to one in Figure 2C) consistent with Figure 2B. However, specific genes were more or less subject- (layer-) variable in all three rhythmic parameters. *PER1,2* and *NR1D2* showed highly variable magnitudes and amplitudes across subjects, while positive regulators *NPAS2*, *ARNTL* were very consistent across subjects. The phases of core clock genes were also much more variable across subjects than across layers (Figure 2C).

Amplitudes of diurnal genes are more variable than phases. The relative variation of amplitudes (viewed using the coefficient of variation) exceeded relative variation of phases both across layers and across subjects (Figure 2D). The 49 genes with different population rhythms between layers (Figure 1E, F) showed high amplitude (Fligner–Kileen test, *P* < 0.0001) and phase (Fligner–Kileen test, *P* < 0.0001) variability across layers in comparison to the 917 genes with indistinguishable rhythms. Core clock genes had remarkably low variability in amplitude and phases across layers consistent with indistinguishable population rhythms we observed across layers (Figure 2D). *NR1D1* was the only exception that showed greater variation in amplitude across layers; it was rhythmic in both layers but with an amplitude just below our cutoff in the epidermis. However, the core clock genes had greater variability in phase across subjects than layers (Wilcoxon signed-rank test, *P* = 0.0005), but with comparable amplitude variability across subjects and layers (Wilcoxon signed-rank test, *P* = 0.2402).

Amplitudes of diurnal genes in the epidermis differ more than in the dermis. We observed previously that epidermal population rhythms had greater amplitudes than dermal rhythms (Figure 1E). In addition, when the variability was quantified in the two layers separately, amplitude variability in the epidermis across subjects exceeded that in the dermis (Figure 2E). Thus, amplitudes in the dermis were smaller but more consistent than amplitudes in the epidermis, which were larger and more variable. Nevertheless, magnitude and phase variabilities were similar in the two layers.

### Predictive biomarkers of internal time in human dermis and epidermis

Finally, we assessed the viability of skin samples to be used for circadian phenotyping. Expression of biomarker genes in the skin can serve as predictors of internal phase of entrainment or chronotype, if a fixed phase relationship between the skin clock and the central SCN clock can be assumed. To predict *internal* time from a single sample, we identified biomarkers among genes expressed in either layer individually (as suggested by the layer-specificity) using ZeitZeiger (40).

**Figure 3.**
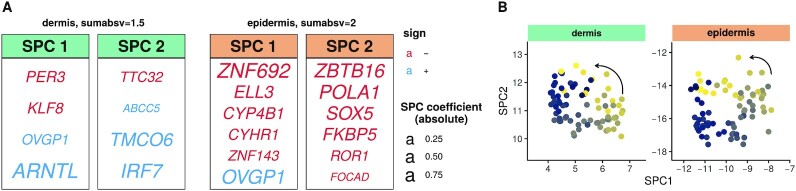
Identification of internal time-telling genes in human dermis and epidermis. (**A**) Predictive biomarkers from human dermis (left panels) and epidermis (right panels) for optimal parameter choice (Supplementary Figure S5A). Genes assigned to SPC1 or SPC2 as well as their coefficients are shown. (**B**) Expression profiles of the predictor genes in each sample in our cohort in the dermis (left) and the epidermis (right) represented in SPC space. Colors indicate the internal time of the subject samples. ZeitZeiger was used to identify biomarkers of internal phase and was run with all ∼11000 expressed genes, separately for the dermis and the epidermis.

A small set of population rhythmic genes accurately predicts internal time. For an optimal parameter choice, 8-12 rhythmic genes (listed in Figure 3A) were sufficient to predict internal time with a median absolute error (MAE) of ∼1.2 h (Supplementary Figure S5A). The ability to infer internal time from a single sample can be seen in the counter-clockwise arrangement of samples projected on the two sparse principal components (Figure 3B). The biomarkers found in the two layers all exhibited robust population diurnal rhythms (Supplementary Figure S5B,C) with the exception of one gene *FOCAD*, which was also rhythmic but with an amplitude below our cutoff. The genes chosen as biomarkers all showed particularly low variability in amplitude, magnitude and phase across subjects according to our analysis in the previous section (Supplementary Figure S5D).

The biomarkers sets contain layer-specific genes, but are depleted of core clock genes. Curiously, the biomarkers for internal time included only two canonical clock genes (*PER3*, *ARNTL*) and that too only in the dermal set. Moreover, the smaller biomarker set for the dermis shared only one diurnal gene (*OVGP1*) with the larger set for the epidermis. The biomarkers in the dermis consisted of genes that were diurnally-rhythmic (at the population level) in both layers. However, the epidermal biomarker set included several genes that were rhythmic only in the epidermis (*POLA1*, *ROR1*, *SOX5*, *ZNF143*). These sets also overlapped poorly with previously published biomarkers for epidermis (*ZBTB16*, *FKBP5*) (19) and dermis (*PER3*, *ARNTL*) (20).

Taken as a whole, our analysis indicates that either dermis or epidermis can be used for phenotyping circadian phase using a small set of mostly skin-specific diurnal genes.

## DISCUSSION

This study aimed to characterize 24 h gene expression rhythms in human skin. Human studies have to cope with the heterogeneity of individuals and their clocks (25), in contrast to circadian gene expression studies in mice (48). We addressed this challenge in several ways: First, our study only included young, healthy subjects with intermediate chronotypes and stable natural sleep-wake rhythms; we nevertheless included male and female subjects to meaningfully describe diurnal rhythms in the population. Second, we corrected sampling times for chronotype differences between subjects (using mid-sleep time on free days after correcting for sleep debt during week days) to present results with respect to internal time; this was only possible due to the chronobiological profiles included in our study design. Third, we structured our characterization to describe (a) the diurnal gene expression on average in a random healthy member of the population and (b) the extent of the deviation of the diurnal gene expression of that random member from the average diurnal expression.

We found ∼1400 genes with population diurnal rhythms in either layer, thus, significantly expanding on the list of known clock associated genes in the skin (19,20). This represents ∼10% of the expressed genes like in most circadian mammalian tissues (6,7) suggesting that our results capture most of the rhythmic genes. This improvement resulted from the higher frequency of sampling (every 4 h) in our study in relation to past studies (18–20). The coarser sampling reduces the power of rhythm detection and degrades estimates of amplitude and phases, which is evident in the poor overlap of 24 h rhythmic genes and poor correlation in amplitudes and phases between our and prior studies. Two-thirds of these genes had indistinguishable rhythms between the layers. On the one hand, this is unsurprising given the physical proximity between the layers. On the other, it is unexpected given the well-documented heterogeneity of the skin (49) and tissue-specificity of circadian programs in physiology (7). The significant inter-subject variability of diurnal gene expression we discovered also makes differences in population rhythms between layers harder to detect, potentially resulting in lower apparent layer-specificity. Core clock gene expression too was consistent between the layers with rare exceptions. Despite this general similarity, a third of the diurnal genes did display rhythmic expression in only one or the other layer. Epidermal diurnal genes were more in number and tended to have larger amplitudes than the dermal diurnal genes (as suggested previously (20)). This might be due to the greater cellular heterogeneity of the dermis (49) or the direct exposure of the epidermis to the external environment.

To quantify the deviations of subjects from the average 24 h rhythm of gene expression, we developed a novel method based on linear mixed models and error propagation. Unexpectedly, the magnitude of diurnal genes overall remained consistent across subjects in each layer, even though it varied across layers. This result supports the claim that 24 h rhythms in humans can be constructed from population sampling, i.e., one sample per individual and thus side-step cumbersome and potentially unethical longitudinal sampling (7,19,50). However, amplitudes and phases of diurnal genes varied more across subjects than layers (in absolute terms and as a fraction of variance). But, the expression of specific diurnal genes were relatively more or less subject-variable. For instance, negative core clock members (*PER1*, *PER2*) had highly subject-variable magnitudes and amplitudes, while positive core clock members (*ARNTL*, *NPAS2*) were the opposite. One consistent feature of the core clock genes was the high subject-variability of their phases in both layers. This might reflect the fact that the MSF_sc_ does not fully account for the chronotype differences between the subjects. Amplitudes generally varied more than phases measured relative to population means across subjects in each layer. Amplitudes of circadian (and diurnal) rhythms are expected to decrease with age (51), but were not previously known to vary more than phases in similarly-aged young subjects. Finally, dermal rhythm amplitudes were smaller but less variable across subjects, while epidermal rhythms possessed larger amplitudes and were more subject-variable. This represents an interesting dichotomy: the dermis might be a better source of circadian biomarkers, but the epidermis might be more indicative of amplitude differences between individuals with a larger dynamic range.

In recent years, a number of novel approaches have been introduced to assess circadian parameters (in particular, circadian phase) in humans based on machine-learning on high-dimensional -omics data (see (52) for a nice review). We therefore explored the suitability of these two skin layers to provide biomarkers to predict internal clock phase from single samples. Similar to our previous work on blood-based circadian phase determination (41), the expression of a small set of 8–12 circadian genes at a single time point was sufficient in either skin layer to predict internal clock phase with a median accuracy of ∼1 h. This accuracy is probably optimistic as it is based on internal cross-validation and a separate validation is necessary to estimate its true accuracy. Even with some loss of accuracy, these biomarkers might be expected to perform as well as biomarker sets previously proposed for the epidermis and dermis (19,20). Genes in biomarker sets must possess consistent magnitudes and amplitudes across subjects in addition to robust rhythmicity in order for the inference from a single sample to be feasible. Our biomarker set for each layer showed the desirable properties of low magnitude and amplitude variability across subjects. This appears to contradict our observation of significant subject variability of circadian gene expression. The resolution lies in the fact that there remains a fraction of circadian genes with low subject-variability that the machine learning finds. Unlike other identified circadian biomarkers for phase (19,20,41,53), the sets discovered in this study were almost devoid of core clock genes. Moreover, most biomarker genes were either rhythmic only in the layer in question or the genes differed significantly in amplitude and/phase from the other layer. This raises the unexpected possibility that biomarker sets involving tissue/layer-specific circadian genes might be better suited for internal phase prediction than core clock genes.

Our results leave open questions that need to be addressed in future studies. Our cohort was small, healthy, young and Caucasian and it is unclear how much our results can be extrapolated to a diverse population. Our inability to find differences between an analysis based on internal and external time was surprising, but is probably due to both the lack of extreme chronotypes in our cohort and insufficiency of 4 h sampling resolution to accurately reflect the ∼6 h range of 95% of human chronotypes; in fact, our cohort has an even smaller chronotype spread of 3.5 h. The estimates of the variability in gene expression across subjects are affected by the small cohort size. In fact, the inter-subject variability subsumes inter-sex variability, which cannot be reliably separated in this small cohort. Variance estimates across just two layers are well defined, but are likely less accurate compared to variance estimates across subjects. The error propagation analysis to quantify variation of rhythm parameters is based on linearization and hence, assumes estimated mixed effects are ‘small’. Moreover, we identified circadian biomarkers for healthy young individuals maintaining natural yet regular sleep schedules. Whether these are also good markers in elderly and sick individuals and those with disrupted sleep schedules, such as shift workers, remains unanswered. It is unknown whether the human skin clock has a fixed phase relationship to or is independent of the central clock (54). If the former, then the identified biomarkers can be used to predict central clock phase, else they would only be able to predict peripheral skin clock phase.

## CONCLUSION

We presented the most complete description to date of the transcriptional output of the circadian clock in two human skin layers. We reported both how average gene expression rhythmically varies in the population and the inherent variability in these average rhythms due to population heterogeneity. Our consideration of internal time in the analysis makes our results applicable to humans regardless of chronotype. Not only does our work provide a comprehensive resource of diurnal gene expression and circadian biomarkers for phase in human skin, but also provides a methodology to describe human circadian rhythms in a population.

## ABBREVIATIONS

SCN: suprachiasmatic nucleus; MSF_sc_: mid sleep time on free days after correcting for sleep debt during week days; FDR: false discovery rate; MAE: median absolute error; GEO: Gene Expression Omnibus; SPC: sparse principal component.

## DATA AVAILABILITY

The gene expression microarray data of dermis and epidermis generated in this study are available at the Gene Expression Omnibus (GEO) database and can be accessed under the accession number GSE205155. The source code is available through GitHub: https://github.com/bharathananth/skin-data-analysis and Zenodo: https://doi.org.10.5281/zenodo.7405300.

## Supplementary Material

lqac097_Supplemental_Files
