# Supplementary material for: Inter-layer and inter-subject variability of diurnal gene expression in human skin
Source: NAR Genom Bioinform. 2022 Dec 31;4(4):lqac097. doi: 10.1093/nargab/lqac097 (PMC9803873; doi:10.1093/nargab/lqac097)
Supplement: lqac097_Supplemental_Files [file lqac097_supplemental_files.zip › NAR-supp-main.pdf]

Inter-layer and inter-subject variability of diurnal gene expression in human skin

Marta del Olmo[1], Florian Spörl[2], Sandra Korge[2,3], Karsten Jürchott[3,4], Matthias Felten[5,6], Astrid Grudziecki[3], Jan de Zeeuw[7], Claudia Nowozin[7], Hendrik Reuter[2], Thomas Blatt[2], Hanspeter Herzel[1], Dieter Kunz[7], Achim Kramer[3], Bharath Ananthasubramaniam[1,8]

bharath.ananthasubramaniam@hu-berlin.de

Supplementary Material

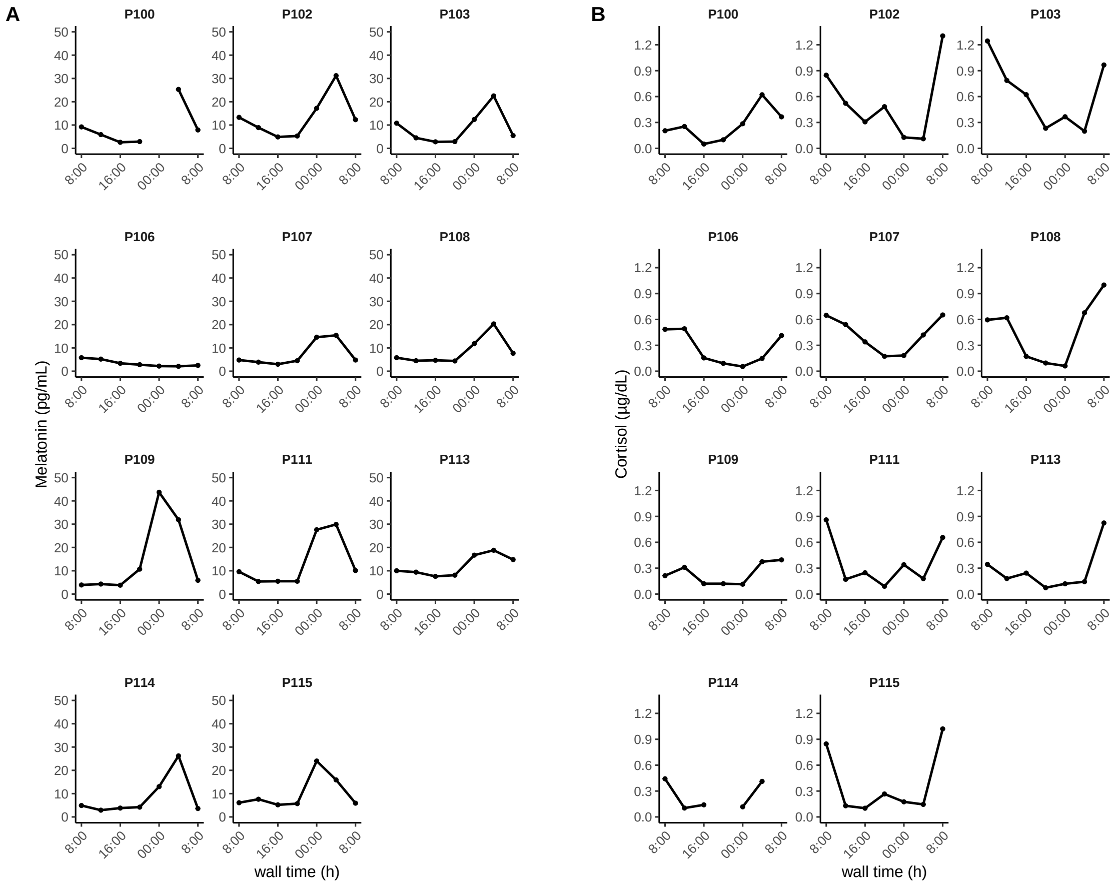

Figure S1: Melatonin and cortisol profiles during the regular entrained routine. A. Individual secretion profiles for melatonin and **B.** cortisol during the study. Melatonin concentrations are indicated in pg/ml and cortisol concentrations in µg/dl. There were a total of one missing melatonin and two missing cortisol samples due to technical problems or insufficient material.

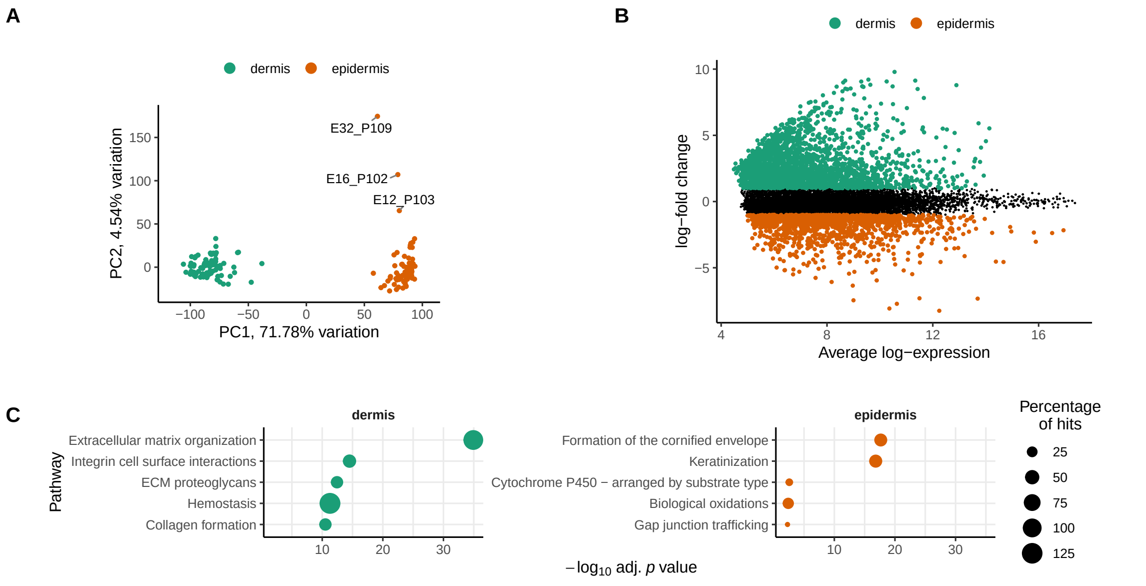

Figure S2: Quality control of the microarray gene expression data. A. Principal component analysis (PCA) of the gene expression data. The two fist principal components (PC) are shown: the first PC separates samples by layer, the second PC separates outliers. The largest outlier (E32_P109) was removed from the analysis. **B.** Mean-Difference plot of $\log_2$ expression in dermis versus epidermis. Each point represents a gene. Green points indicate genes that are differentially expressed in dermis (log-fold change $>$ 1 in dermis compared to epidermis at a FDR $<$ 0.05); orange points indicate genes that are differentially expressed in epidermis (log-fold change $<$ -1 in dermis compared to epidermis at a FDR $<$ 0.05); black points indicate genes that are not differentially expressed in dermis compared to epidermis. **C.** Reactome pathway enrichment analysis of the differentially expressed genes in dermis (1976, green) and epidermis (1164, orange) tested against the background of all 11578 expressed genes. Only pathways containing more than 20 genes per set at a p value $<$ 0.05 are shown.

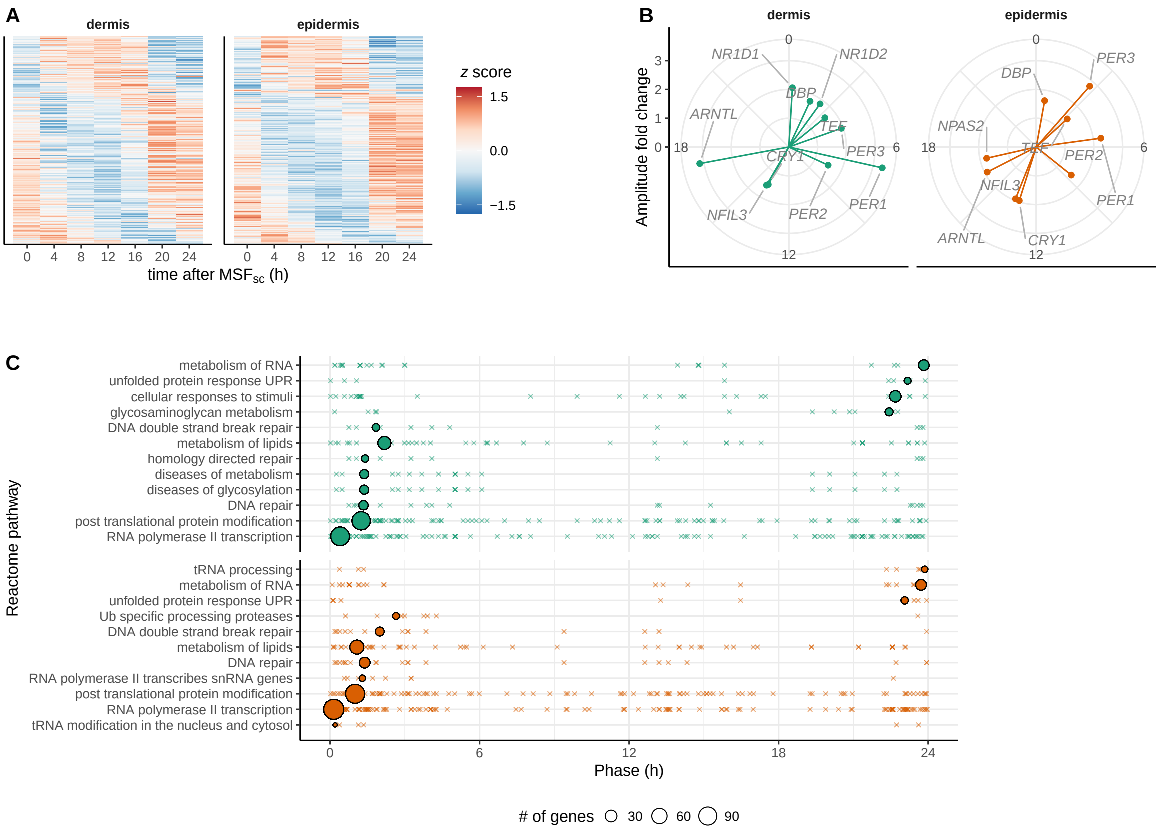

Figure S3: Population diurnal gene expression in healthy human skin. A. z score-normalized, acrophase-ordered expression heatmap of the diurnal genes from human dermis (left) and epidermis (right). **B.** Expression profiles of diurnal core clock genes in human dermis (left) and epidermis (right). Arrow direction represents phase (expressed as peak time, in hours, after MSF$_{sc}$) and arrow length depicts peak-to-trough fold change amplitude. **C.** Summary of phase-clustered diurnal Reactome pathways ($q < 0.05$) in dermis (green) and epidermis (orange) as determined by PSEA against a uniform background distribution (sets containing fewer than five diurnal genes were excluded from the analysis).

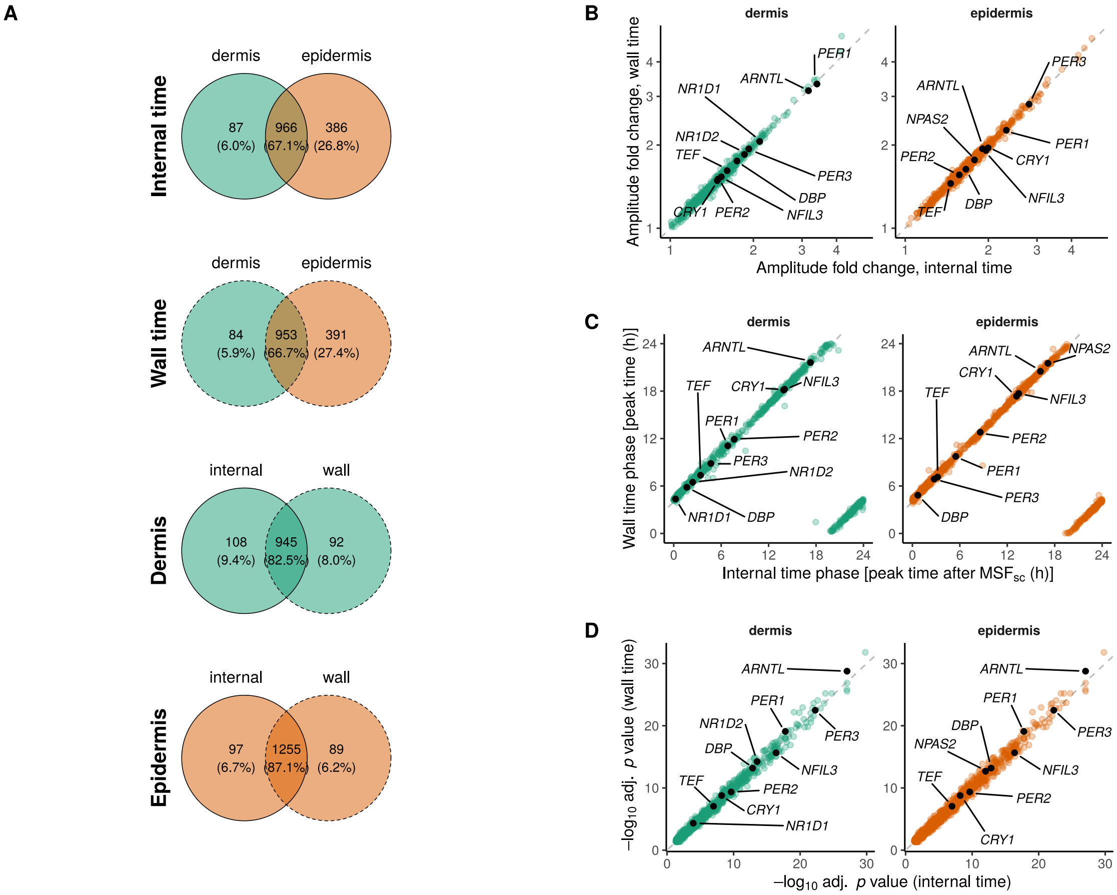

Figure S4: Population diurnal rhythms in human skin are similar when time is not adjusted for chronotype differences. A. Venn diagram visualization of the number of genes identified as diurnal in dermis (green) vs. epidermis (orange) and in the analysis using internal time (i.e., after correcting for chronotype differences, solid line) or wall time (dashed line). **B.** Amplitude correlation of genes identified as rhythmic with the internal time analysis compared to external time analysis. **C.** Phase correlation of diurnal genes identified with the internal time analysis compared to external time analysis. Phase with respect to internal time was defined as peak time after MSF_{sc}, while phase with respect to wall time is the peaking time of the respective gene without correction. **D.** Benjamini Hochberg-adjusted *p* value correlation of rhythmic genes identified with internal vs. external time analysis.

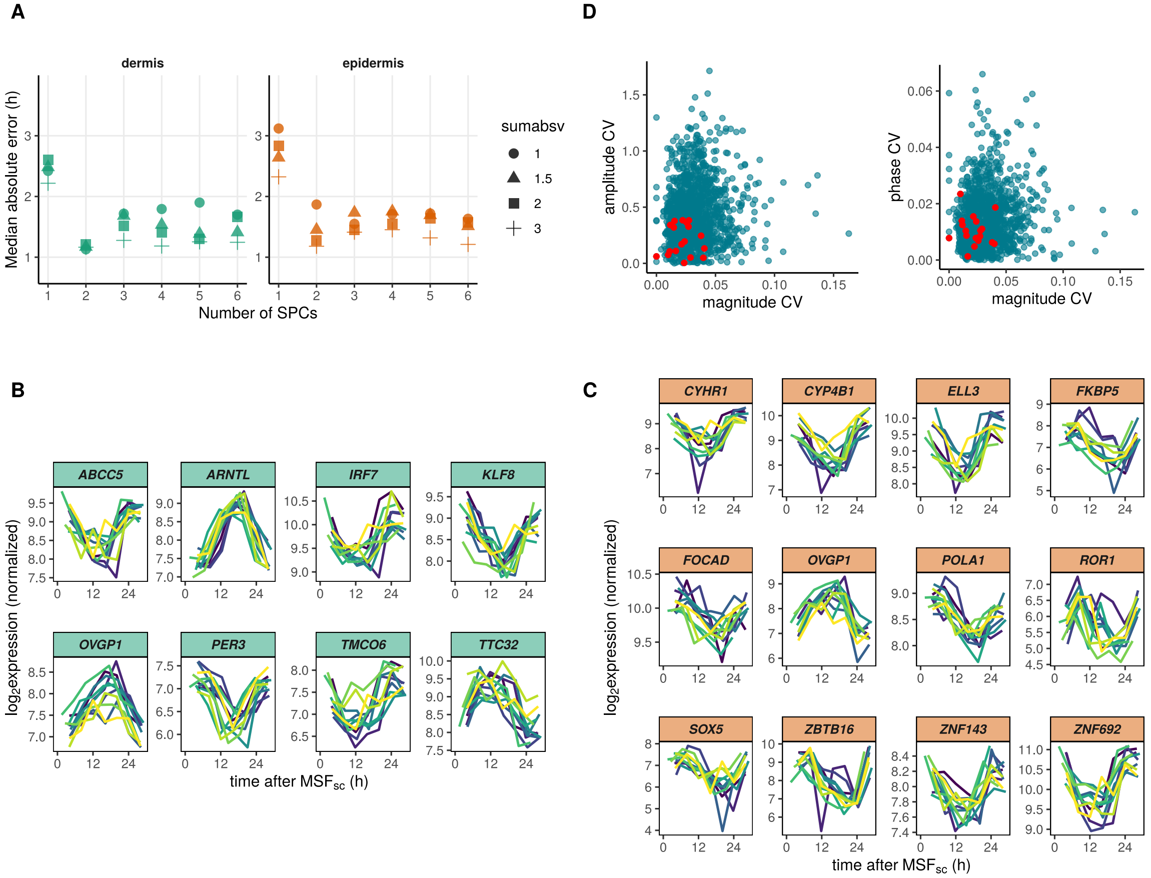

Figure S5: Predictive biomarkers of internal time in human dermis and epidermis. A. Median absolute error of the internal-time prediction on cross-validation (see Materials and Methods for details) as a function of the two main parameters of `ZeitZeiger`, `sumabsv` and `nSPC`. **B.** Expression profiles of the time-telling genes in dermis (green) and **C.** epidermis (orange) for optimal parameter choice of `sumabsv` and `nSPC`. Colored lines represent the time series in different subjects. `ZeitZeiger` was run with all $\sim$ 11000 expressed genes, separately for dermis and epidermis. **D.** Quantification of magnitude, amplitude and phase variability of diurnal genes across subjects. Predictive biomarkers are shown in red.

Table S1: Information and sleeping schedules of the healthy subjects who participated in the study. Chronotypes were estimated from sleep schedules as the mid-sleep time on free days after correcting for sleep debt (MSF_{sc}) [1,2].

Subject	Sex	Age in year of study (2011)	Bed time work days	Sleep time work days	Min fall asleep work days	Wake up time work days	Min wake up work days	Alarm work days?
P108	male	27	22:45	23:00	20	6:30	15	Y
P100	male	20	23:00	23:00	7.5	7:00	5	Y
P113	male	26	23:30	0:00	30	8:30	10	Y
P106	male	29	23:00	23:15	15	6:52	7.5	Y
P102	male	22	23:00	23:00	10	7:30	0	Y
P109	male	27	23:00	23:00	5	8:00	0	Y
P103	female	23	0:00	0:25	25	7:30	9	Y
P107	female	24	23:00	23:00	15	6:00	5	Y
P111	female	28	0:00	0:00	5	8:00	15	Y
P114	female	25	23:30	23:30	5	8:30	5	Y
P115	female	30	22:00	22:15	5	5:20	5	Y

Subject	Bed time free days	Sleep time free days	Min fall asleep free days	Wake up time free days	Min Wake up time free days	Alarm free days?	Corrected mid sleep time
P108	23:00	23:15	15	6:30	30	N	02:58
P100	0:00	0:00	5	8:00	15	Y	04:00
P113	1:00	1:15	20	9:30	15	Y	05:28
P106	23:30	23:45	15	9:15	60	Y	03:50
P102	0:00	0:00	10	8:00	10	N	04:11
P109	0:00	0:00	5	8:30	30	N	04:26
P103	0:00	0:00	25	7:30	5	Y	03:36
P107	0:00	0:00	15	7:30	5	N	03:34
P111	2:30	2:30	5	11:00	30	N	06:34
P114	23:30	23:30	5	8:30	5	N	04:00
P115	0:00	0:15	5	8:30	30	N	03:58

Table S2: List of differentially-expressed genes in each skin layer. External .xlsx file.

Table S3: List of genes with population diurnal gene expression in each layer. External .xlsx file including phase and amplitude values estimated from prior skin studies [3–5].

Table S4: Estimated variability of diurnal gene expression parameters across subjects and layers. External .xlsx file.

References

[1] Roenneberg, T. *et al.* Epidemiology of the human circadian clock. *Sleep medicine reviews* **11**, 429–438 (2007).

[2] Vetter, C., Winnebeck, E. C. & Roenneberg, T. Asking the clock: How to use information from questionnaires for circadian phenotyping. In *Circadian Clocks*, 79–85 (2021).

[3] Akashi, M. *et al.* Noninvasive method for assessing the human circadian clock using hair follicle cells. *Proceedings of the National Academy of Sciences* **107**, 15643–15648 (2010).

[4] Wu, G. *et al.* Population-level rhythms in human skin with implications for circadian medicine. *Proceedings of the National Academy of Sciences* **115**, 12313–12318 (2018).

[5] Wu, G. *et al.* A population-based gene expression signature of molecular clock phase from a single epidermal sample. *Genome medicine* **12**, 1–12 (2020).
